# Consensus on acute behavioural disturbance in the UK: a multidisciplinary modified Delphi study to determine what it is and how it should be managed

**DOI:** 10.1136/emermed-2023-213335

**Published:** 2023-09-22

**Authors:** Christopher Humphries, Anthony Kelly, Aws Sadik, Alison Walker, Jason Smith

**Affiliations:** 1 Centre for Cardiovascular Science, Queen's Medical Research Institute, Edinburgh, UK; 2 Emergency Department, University Hospitals Plymouth NHS Trust, Plymouth, UK; 3 Centre for Academic Mental Health, Population Health Sciences, Bristol Medical School, Bristol, UK; 4 Avon & Wiltshire Mental Health Partnership NHS Trust, Bath, UK; 5 West Midlands Ambulance Service University NHS Foundation Trust, West Midlands, UK; 6 Emergency Department, Harrogate and District NHS Foundation Trust, Harrogate, UK; 7 Academic Department of Military Emergency Medicine, Royal Centre for Defence Medicine (Research and Academia), Birmingham, UK

**Keywords:** toxicology, Forensic Medicine, psychiatry

## Abstract

**Background:**

Acute behavioural disturbance (ABD) is a term used in law enforcement and healthcare, but there is a lack of clarity regarding its meaning. Common language should be used across staff groups to support the identification, prioritisation and delivery of care to this group of patients. The terminology currently used is inconsistent and confusing. This study aimed to reach a consensus on the criteria for identification and management of ABD, and to agree when other care pathways or guidelines might be more appropriately used.

**Methods:**

A modified Delphi study with participation from stakeholder organisation representatives was conducted in January–April 2023 online. In round 1, statements were generated by participants in response to broad questions. Participants then rated their level of agreement with statements in subsequent rounds, with statements achieving a consensus removed for inclusion in the final derived consensus statement. Non-consensus statement responses were assessed for stability.

**Results:**

Of 430 unique statements presented for rating, 266 achieved a consensus among 30 participants representing eight stakeholder organisations. A derived consensus statement was generated from these statements. The median group response to statements which failed to achieve a consensus was reliable (Krippendorff’s alpha=0·67).

**Conclusions:**

There is a consensus across stakeholder organisations that ABD is not a separate entity to agitation, and guidance should instead be altered to address the full range of presentations of agitation. While the features of concern in this severely agitated group of patients can be described, the advice for recognition may vary depending on staff group. Criteria for recognition are provided and potential new terminology is described.

**WHAT IS ALREADY KNOWN ON THIS TOPIC**

- Acute behavioural disturbance (ABD) is a term used in the UK to describe presentations of severe agitation, distress and signs of physiological deterioration of unknown cause.
- There is a lack of consensus on ABD recognition criteria in policing and healthcare, whether there is value in the use of ABD terminology and when alternative guidelines may be more appropriate than ABD guidance.
- There is a lack of validated UK criteria on which to base management guidelines. This has led to many guidelines using research published on ‘excited delirium’– a condition which is not recognised in the UK.

**WHAT THIS STUDY ADDS**

- This study is the first to bring together stakeholder organisations in the UK to achieve a consensus on the value of ABD terminology, criteria for recognition and response to severely agitated patients at greatest risk, and when the use of current guidelines on ABD is most appropriate.

**HOW THIS STUDY MIGHT AFFECT RESEARCH, PRACTICE OR POLICY**

- This study provides a clear consensus that ABD is not a separate entity to agitation, but there are criteria which can be used to identify agitated patients at greatest risk of poor outcomes. Specific terminology should be used to identify this group and provide a common language regarding prioritisation and management strategies. Consideration should be given to using new terminology such as ‘red-flag agitation’ to describe the most severely agitated patients at the greatest risk of physical health emergency.

## Introduction

Acute behavioural disturbance (ABD) is the term used by emergency care providers in the UK to describe ‘an altered physiological and psychological state’.^1^ There may be unrecognised life-threatening illness leading to the behavioural disturbance, and management is complicated by the potential increased risk of restraint-related death.

Use of the term ABD has been recommended by several coroners’ inquests in an effort to ensure that standardisation of terminology promotes the best possible care being provided to all patients, some of whom are among the most clinically challenging cases for providers to manage.^2^ However, variation still persists among different organisations in the UK, and there is a lack of clarity around the use of the term ABD as a presentation of severe agitation, rather than a diagnosis or syndrome.^3–6^ This also makes it challenging to hold organisations to account and drive improvement in patient care.^7^

Use of the term ABD is further complicated by blurred boundaries with excited delirium (ExD), which is a term predominantly used in North America and which (as cogently described by McGuinness and Lipsedge in their 2022 paper) is widely considered problematic, with no proven pathological basis.^8^ There have been calls to replace the term ABD with a new descriptor due to concerns about conflation with ExD, but until the patient group in question is clearly defined, any new descriptor faces the same risk of conflation due to a lack of evidence on which to base advice regarding the identification, risk stratification and management of patients presenting with severe agitation.^9^

Several UK organisations have produced guidelines in an attempt to meet their responsibility to provide care for people with behavioural change, but the challenge of identifying people at risk of deleterious outcomes still remains.^1 3 4 6^ It is clear that until a consensus is achieved, from which an evidence base can be generated, there will continue to be concerns that consequent guideline application (including use of force and sedation) may be racially biased.^7^ A lack of a UK-wide consensus definition adversely affects the ability of the services and systems involved to consistently apply guidance, and prevents standardised identification of the cohort of patients on whom UK research should be based, with the aim of improving care.

Establishing objective criteria for recognition of ABD is challenging, as the requirement for patients to be exhibiting an altered physiological state to identify ABD is often at odds with the inability of clinical staff to be able to measure physiological parameters due to the level of agitation. Concerns have been raised that, as a consequence, police and clinicians may be unsure of when to recognise a presentation of ABD or may apply ABD guidance to all agitated patients. Additionally, it is unclear who is responsible for recognising ABD; and whether it is a law enforcement or healthcare term, a presentation or a diagnosis. This has led to guidelines being produced by multiple professional bodies, with different objectives and perspectives.

This study aimed to reach a consensus on the criteria for identification and management of ABD, and to agree when other care pathways or guidelines might be more appropriately used.

In reaching a consensus, it is hoped that there will be benefits for service users, as appropriate care pathways are followed more consistently and with reduced subjectivity; for staff, as clarity around clinical decision-making improves, and for future researchers, as clear ABD criteria are established.

## Methods

To achieve a consensus, a modified Delphi study was undertaken with representatives from key stakeholder organisations. Recruitment took place in November–December 2022, and the study took place over three rounds in January–April 2023.

### Patient and public involvement

As presentations of ABD are primarily and formally recorded at inquest, but are not captured in UK clinical coding, it was not possible to identify people with lived experience of the presentation. To ensure that the study outcomes were relevant to patients, we invited comment on the aims and design of the study from the relatives of a patient who died after presenting with ABD, and also sought views from the NHS Race and Health Observatory on the objectives and design of the study. We were asked (as a standard requirement prior to the consultation being agreed) to explicitly state in any publication that participation in consultation does not equate to endorsement of any study or its outcomes. These parties were not involved in the recruitment or conduct of the study.

Study participant organisations will receive a report of the study findings for comment and action within their remit areas.

### Participant selection

To minimise selection bias, invitations were issued to participant organisations identified as key stakeholders in a Royal College of Psychiatrists report, of which our study group were not authors.^9^ Based on our external consultation, we also invited participation from toxicologists and two patient advocacy organisations. The organisations are shown in table 1.

**Table 1:** Organisations invited to participate in the Delphi study and outcomes of those invitations

Sector	Organisation	Outcome
Police	National Police Chiefs’ Council	Participated
Judicial/coronial	Coroners’ Society	Declined (due to perceived implications for court process)
	Office of the Chief Coroner	Email acknowledged, but no participants entered
	Royal College of Pathologists	Email acknowledged, but no participants entered
Custodial	Faculty of Forensic and Legal Medicine	Participated
	UK Association of Forensic Nurses and Paramedics	Participated
Ambulance care	Association of Ambulance Chief Executives	Participated
Emergency care	Faculty of Intensive Care Medicine	Participated
	Royal College of Emergency Medicine	Participated
Mental health	Royal College of Psychiatrists	Participated
	Mind (advocacy group)	Declined (due to lack of relevant expertise)
Toxicology	National Poisons Information Service	Participated
	European Network of People who Use Drugs (advocacy group)	No acknowledgement of attempts to contact

Organisations received up to five phone calls (if a phone number was provided) and five emails in an effort to establish contact and to confirm if they were considering the invitation. Organisations were permitted to delegate participation to professional advisory group members. Eight organisations submitted participants, two declined to participate, two acknowledged contact but did not submit participants without offering a reason and one did not respond to attempts to contact them.

Organisations were invited to submit up to five experts for participation. The Consensus on Acute Behavioural Disturbance in the UK (CABDUK) study group were not involved in the selection of these experts and were explicitly excluded from participation.

Criteria for expertise were suggested as typically being authorship of a publication related to ABD, UK committee-level work on ABD or expert witness (in UK Court) work relating to ABD. However, the requirement was not made absolute as each organisation was likely to have a different perspective on expertise they wished to see represented.

### Study process

To maximise expert engagement from across the UK, the study was conducted electronically using Google Forms. Participant anonymity was maintained for the duration of the study, though participants from within organisations may have been aware of each other’s participation.

Each round launched with an email. Participants who had not submitted responses by half-way through the allotted time received up to two phone calls. If this failed to establish contact, a text message and a further email were sent. This process was repeated in the final few days of each study round. No financial incentives or rewards were offered for completion. Non-participation in prior rounds did not preclude participation in subsequent rounds.

The study was conducted in three rounds. Responses to eight broad questions offered in round 1 were reviewed for uniqueness and duplicates were removed by study group consensus. In round 2, the list of unique statements was presented to participants for agreement/disagreement using a 7-point Likert scale. Statements were removed if they achieved positive or negative consensus to enter the derived Delphi statement.

In round 3, remaining statements were re-presented to participants for voting with the median group score and the participant’s personal prior rating if they had participated in the previous round. Statements achieving a positive or negative consensus then entered the derived Delphi statement.

### Definition of consensus

There is no established definition for a Delphi consensus.^10^ We defined a consensus as 75% agreement or disagreement, as by convention many Delphi studies appear to use either 70% or 80% as consensus values. This value was declared in advance in the participant information.

Due to the study including participants from both healthcare and law enforcement backgrounds, and the potential for questions requiring specific clinical or policing expertise, participants were allowed to decline to respond to a question if they felt they had insufficient expertise in that area.

### Generation of broad statements

Eight broad questions were designed by the authors to generate participant statements covering the major issues identified with current application of ABD terminology:

- A lack of clear criteria to enable police officers to recognise ABD.
- A lack of clear criteria to enable healthcare staff to recognise ABD.
- A lack of clear understanding of when alternative guidelines might be more appropriately used than ABD guidelines.
- A lack of a clear understanding whether ABD terminology or guidance is applied across a spectrum of severity.

The eight questions are shown in table 2. Timing thresholds for the spectrum of severity questions were based on UK ambulance service response targets.

**Table 2:** Broad questions presented to participants in round 1 to support the generation of statements for voting

Question number	Question text
1	What features should UK police officers be advised are required to recognise a presentation of acute behavioural disturbance (ABD), and therefore to apply national guidance?
2	What observable features should UK custody healthcare staff, ambulance services and EDs be advised are required to recognise a presentation of ABD, and therefore apply national guidance, if clinical monitoring or clinical investigations cannot be safely achieved?
3	What clinical examination findings (eg, during assessment or examination by a clinician–nurse, emergency medical technician, paramedic, doctor or advanced clinical practitioner) should lead to UK custody healthcare staff, ambulance services and EDs recognising a presentation of ABD, and therefore apply national guidance, if it was not recognised prior to this?
4	There have been concerns raised that ABD guidance may be misinterpreted as applicable to all agitated people. What subsequent or additional information should lead to UK custody healthcare staff, ambulance service and ED providers ceasing to manage a case using ABD guidance and instead using alternative guidance? (moving from guidance recommended for ABD presentations to instead using other clinical guidelines for patients, for example, presenting with mental health problems)
5	For patients who are breathing and conscious: What features following a presentation of ABD necessitate emergency healthcare provider input? (within 20 min)
6	For patients who are breathing and conscious: What features following a presentation of ABD require urgent healthcare provider input? (within 2 hours)
7	For patients who are breathing and conscious: What features following a presentation of ABD permit non-urgent healthcare provider input? (within 4 hours)
8	For patients who are breathing and conscious: What features following a presentation of ABD suggest that observation in police custody would be safe? (care provided solely by custody healthcare staff)

### Analysis

Statistics support was provided by the University of Edinburgh Epidemiology and Statistics Core.

For statements which did not reach a consensus in rounds 2 and 3, reliability of median group responses was assessed using Krippendorff’s alpha.^11^ This was done using the kripp.alpha function in RStudio V.2023.03.0.386 with 95% CIs obtained by bootstrapping.

## Results

### Overall Delphi process

**Figure 1:**
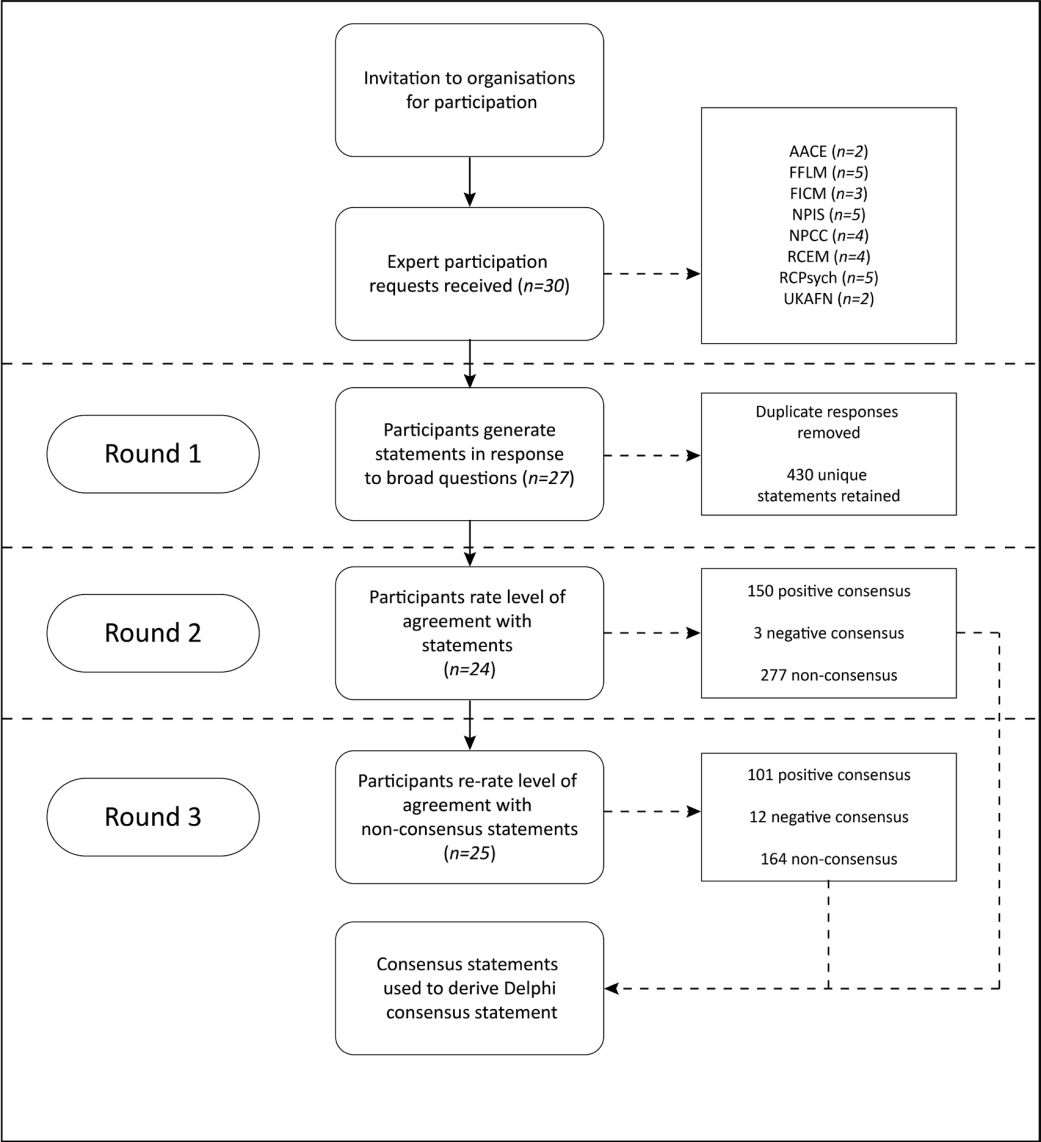
Study flow diagram. AACE, Association of Ambulance Chief Executives; FFLM, Faculty of Forensic and Legal Medicine; FICM, Faculty of Intensive Care Medicine; NPCC, National Police Chiefs’ Council; NPIS, National Poisons Information Service; RCEM, Royal College of Emergency Medicine; RCPsych, Royal College of Psychiatrists; UKAFN, UK Association of Forensic Nurses and Paramedics.

Figure 1 describes the overall process of the Delphi.

### Response to broad statements

Round 1 (broad statements) generated 430 unique statements for voting once duplicates were removed. Care was taken to leave apparent duplicate statements when subtle differences in expression might have affected statement interpretation.

### Consensus statements

Round 2 saw 150 statements achieve a positive consensus, 3 statements achieve a negative consensus and 277 statements fail to achieve a consensus.

Of the 277 statements entering round 3, 101 achieved a positive consensus, 12 achieved a negative consensus and 164 statements fail to achieve a consensus.

Assessment of the reliability of median group responses for statements which did not reach a consensus in round 2 or round 3 gave a Krippendorff’s alpha of 0.67 (95% CI 0.58 to 0.76), which by convention allows a tentative conclusion of rating reliability to be drawn.^12^

The list of statements which achieved/did not achieve a consensus is provided as online supplemental table 1.

10.1136/emermed-2023-213335.supp1 Supplementary data

### Derived Delphi consensus statement

The views of the CABDUK Delphi participant group are given in box 1.

Box 1 **The derived Consensus on Acute Behavioural Disturbance in the UK Delphi consensus statement**

**Regarding recognition of acute behavioural disturbance (ABD) by first responders**

1. All first responders should understand that ABD does not denote a specific diagnosis.
2. For first responders, the triad of being hot to touch (tactile hyperthermia), exhibiting constant or near-constant activity, and extreme agitation or aggression should be the focus of recognition, as the majority of other described signs and symptoms arise as a consequence of these.
3. The triad in (2) includes features which are less vulnerable to racial and gender stereotyping.
4. Features for recognition of ABD may be different depending on professional role (eg, call handler scripts can identify features to prompt responding officers to consider signs of ABD).
5. Individual features are insufficient for recognition of ABD. The person should be exhibiting more than one consensus feature. However, the presence of all consensus features is not required.
6. Features which may be present in presentations of ABD and identified by first responders include:
  - The person is hot to touch.
  - The person is sweating profusely.
  - The person is exhibiting constant (or near-constant) physical activity.
  - The person is unable to sit or stand still.
  - The person is exhibiting extreme agitation.
  - The person is exhibiting extreme aggression.
  - The person’s presentation of aggression or hostility may appear atypical.
  - The person appears disoriented (to place/person/time).
  - The person is exhibiting bizarre behaviour.
  - The person does not respond to other people present.
  - The person is exhibiting exceptional strength.
  - The person is constantly resisting restraint, or is restrained for 15min without resolution or de-escalation.
  - The person does not become calmer with verbal de-escalation or restraint.
  - The person has a raised heart rate.
  - The person’s behaviour is not explained by other factors.
7. Phrases such as ‘superhuman strength’ should not be used.
8. The following features should not be used to identify ABD:
  - Attraction to mirrors or glass.
  - Destruction of mirrors or glass.
9. All first responders should understand that patients presenting with rapid physical deterioration and agitated behaviour should have a focus on management of their physical health.
10. People recognised as presenting with ABD should have a focus on physical health monitoring for evidence of deterioration, use of de-escalation techniques and early transportation to a healthcare facility if there is evidence of deterioration.
11. Police officers should have a high index of suspicion for recognising ABD, and therefore a low threshold to divert to a healthcare provider rather than a custody setting.
12. Live feed from a police officer’s body-worn video to specifically trained individuals in force control rooms may be helpful.
13. All police custody staff should be trained to recognise a patient who is at high risk of deteriorating early.

**In settings with healthcare provision (custody suites, ambulance services or EDs)**

1. Clinical staff have additional skills, training and potential opportunities to undertake patient assessment which may lead to a better understanding of the cause of the presentation of ABD, or negate the need to use the term.
2. Healthcare professionals have a responsibility to differentiate the cause of the patient’s presentation.
3. Staff should:
  - Have plans regarding the management of patients presenting with ABD until emergency care has arrived, including de-escalation.
  - Have access to readily available emergency equipment in environments which may be required to care for people presenting with ABD.
  - Be aware that restraint may worsen a patient’s condition.
  - Understand that the combination of physical health deterioration and behavioural disorganisation is likely to need emergency medical care.
4. Observable features leading to recognition of a presentation of ABD should have more detail for healthcare staff.
5. The triad of being hot to touch (tactile hyperthermia), exhibiting constant or near-constant activity, and extreme agitation or aggression should be the focus of recognition, as the majority of other described signs and symptoms arise as a consequence of these.
6. Individual features are insufficient for recognition of ABD. The person should be exhibiting more than one consensus feature.
7. When recognising a presentation of ABD, there should be a focus on objective findings, such as:
  - Hot to touch/sweating/hyperthermia/removal of clothing.
  - Constant or near-constant physical activity.
  - Severe agitation.
  - Confusion/disorientation.
  - High levels of anxiety.
  - Hypervigilance/fearfulness/panic/paranoia.
  - Tachypnoea.
  - Tachycardia.
  - Hypertension.
8. Additional features which may lead to the recognition of a presentation of ABD by healthcare staff include:
  - Findings on clinical examination:
    - Being unable to obtain observations due to agitation—BP, pulse, pulse oximetry, pupil size.
    - Evidence of autonomic dysfunction.
    - Does not appear to tire, agitated and not interacting, or psychomotor agitation.
    - Exhibiting exceptional strength.
    - Does not understand verbal commands.
    - Psychological distress with the potential to harm themselves or another person.
    - Disinhibited or violent behaviour.
    - Aggression with no identifiable reason.
    - Features of psychosis (eg, hallucinations).
    - Delirium.
    - Sympathomimetic toxidrome.
  - The circumstances of the presentation:
    - Exhibiting behaviour reported to not be normal for them.
    - The features had a sudden onset.
    - Failure of de-escalation techniques.
    - Constant resistance to restraint, or restraint for 15 min without resolution or de-escalation.
    - Sustained non-compliance with police or ambulance staff.
    - Hypoglycaemia has been excluded.
    - Likely to have ingested stimulant drugs.
    - Increased pain tolerance.
    - Acute deterioration.
    - An ongoing need for sedation at higher doses than would typically be expected.
9. The following features should not be used to identify ABD:
  - Attraction to mirrors or glass.
  - Destruction of mirrors or glass.
  - Difficult to palpate a radial pulse.
  - Intolerance to light.
  - Vacant expression.
10. When professionals believe the ABD criteria are met, the person should always be moved to an ED for assessment, as an emergency.

**Regarding the applicability of ABD guidelines**

1. It is not helpful for healthcare staff to have separate guidance for ‘agitation’ and ‘ABD’. Rather, a history should be taken and assessment of the level of agitation should be made. Treatment should be based on the level of agitation and clinical risk. However, this should not be understood to mean that there is no role for the application of guidance currently provided for presentations of ABD.
2. The consensus group do not agree that there is no definition of ABD. The features of concern can be described.
3. As ABD is not a diagnosis, national guidance should be directed towards the safe care of any acutely distressed person.
4. Evidence of rapid and significant physical or physiological deterioration along with agitation or confusion should lead to urgent clinical assessment and management. Other descriptors are not specific.
5. No single sign or symptom should be used in isolation to identify this presentation. The clinical history and preceding events should be used to build a clinical picture.
6. The person does not need to be exhibiting all consensus features, and assessment should not be limited to ‘objective measures’ such as vital signs and blood tests.
7. Regardless of the terminology used, restraint and sedation should always be a last resort. The first response to any acutely distressed or agitated person should be non-physical approaches, including de-escalation.
8. It is hoped that a focus on appropriate recognition of ABD will prevent guidelines for ABD being applied to all agitated people.
9. ABD is not a diagnosis, and recognition of a presentation of ABD does not stop other clinical guidelines becoming relevant.
10. If a likely diagnosis or condition is present, then that should be treated, but there may be a role for using ABD guidance in conjunction with other relevant clinical guidelines.
11. The decision to move to alternative clinical guidance is not the exclusive domain of emergency departments.
12. If the person’s baseline state cannot be determined from them sharing their personal experience, collateral history or medical records, the default should be to assume the abnormal behaviour is acute.
13. Using other guidance may be more appropriate if:
  - It is identified that the patient is struggling to breathe.
  - The person is able to communicate their experiences.
  - The person responds to verbal de-escalation.
  - The person engages in >60 s of consistent verbal communication, or focuses aggression at an individual, demonstrating perception of environment and persons around them.
  - Signs or symptoms of physiological disturbance are not identified on healthcare staff assessment.
  - Additional information becomes available (eg, psychiatric history identifies known schizophrenia) to which the person’s presentation can be attributed appropriately.

**Regarding patients who are breathing and conscious with a suspected presentation of ABD**

1. Healthcare provider input should be available as an emergency (within 20 min) if:
  - There is a triad of tactile hyperthermia, extreme agitation or aggression, and constant or near-constant activity.
  - The person is restrained for 15 min without resolution or de-escalation.
  - There is sudden cessation of resistance, or the person suddenly becomes quiescent.
  - There is a history of syncope or pre-syncope, seizures or chest pain.
  - Features are identified which may be present in ABD or suggest a higher degree of risk, such as:
    - Constant or near-constant activity.
    - Persistent or extreme agitation or aggression.
    - Tactile hyperthermia or sweating profusely.
    - A sudden change in rate or depth of breathing.
    - The person is requiring medication for agitation.
    - The person cannot be calmed with de-escalation techniques.
    - Continued use of force with a high degree of resistance.
    - Clear indications of key drug use (eg, cocaine, other stimulants, phencyclidine, lysergic acid diethylamide).
    - There is an inability to safely manage the risk to self or others.
    - The person is a risk to themselves or others.
    - Hyperkalaemia, raised lactate or acute kidney injury are identified on blood tests.
    - A cause for the person’s presentation cannot be identified.
  - Features are identified on examination:
    - Physiological derangement.
    - An abnormality identified in primary survey.
    - Abnormal vital signs, blood glucose or ECG.
    - Confusion.
    - Features of psychosis.
    - Bizarre behaviour or thoughts.
    - Clonus.
    - Significant physical injuries.
    - Exhibiting behaviour reported to not be normal for them.
2. All people presenting with ABD require an emergency response.
3. The same criteria should be used as for anyone with acute agitation.
4. A person presenting with ABD cannot wait 2 hours for assessment.
5. If a person initially presented with high-risk features but has subsequently improved, then non-emergency healthcare response may become appropriate.
6. If a person’s behaviour has returned to normal and the features identified in (37.f) are excluded on clinical examination, a subsequent non-urgent response is possible.

**Regarding observation in police custody**

1. Observation in police custody would only be safe following diagnosis of the cause of the ABD and return of temperature, pulse, RR and other clinical parameters to acceptable levels having been monitored in a setting with full resuscitation facilities.
2. The person should have decreasing levels of agitation, and this should be manageable with simple measures.
3. The person should be coherent and able to converse giving rational responses.

## Discussion

### Applicability

This study is the first in the UK to bring together stakeholder organisations across the entire patient journey to clearly establish what ABD is understood to mean in a UK setting, and expectations around recognition and care. The use of formal research methodology serves to increase the credibility of the conclusions. The study authors had no role in selecting each organisation’s representatives, and the heterogeneity of the participant organisations should provide assurance that a range of views have been represented. Some of the statements closely mirror current guidance from different UK groups.

A consensus has been achieved across professional groups in what has historically been a contentious area. However, it is important to interpret the consensus statements in the UK’s social context and avoid simplistic narratives or the application of these statements without thought to potential harms.

### Key findings

From the study consensus, it is clear that ABD is not, in the UK, considered a diagnosis or syndrome, and refers to a presentation of an individual in a state of severe agitation, with numerous potential causes. There was support for integrating current guidance for agitation and ABD to provide guidelines which are applicable to the entire spectrum of presentations of agitation. It was felt that the features of concern in ABD can be described, and that (while not applicable to all agitated people) ABD guidance addressed a clear need which was not met by other agitation guidelines.

Importantly, there are clear differences in expectations between first responder and healthcare provider use of ABD terminology. First responder recognition is focused on the identification of people for whom physical health management should be prioritised, while the subsequent healthcare focus is informed by a recognition of ABD as a very severe presentation of agitation, with multiple potential causes, some of which may be life-threatening. Recognition of this level of agitation by healthcare providers allows concurrent use of ABD guidance and any other relevant guidelines.

There is a clear desire to limit the use of some features historically associated with ExD (eg, 8.a—glass attraction) but a clear consensus that in spite of stereotyping concerns, some features are important in identifying patients presenting with agitation who are at greatest risk (eg, 21.b.viii—increased pain tolerance). In places, this has led to potentially contentious statements, such as recommending the avoidance of phrases such as ‘superhuman strength’ (identified as a criterion disproportionately applied to people from black ethnic groups) while simultaneously identifying ‘exceptional strength’ as a feature which may be identified in presentations of ABD.^13^

This study is also the first to identify criteria for return to police custody following a presentation of ABD, opening the door to a more collaborative approach between emergency care providers and custody suites.

### The future of ABD terminology

Historically in the UK, the use of ABD as a term has been promoted by learning from coroners’ inquests held after deaths, in which it has been identified that improved recognition of the risks to health has the potential to prevent future deaths and provide a common language between emergency services.^2^ This study consensus has recognised that ABD is considered a state of severe agitation, but with features which suggest significant risk to physical health. However, the clear consensus that it is not helpful to separate guidance on ABD and agitation raises the question of the value of using ABD terminology in policing and healthcare as opposed to a new term to identify patients presenting with agitation who are at greatest risk of experiencing a time-critical or physical health emergency. This is particularly important given concerns from advocacy organisations regarding how terminology which emerged from historical use of ExD may be applied.^14^

**Figure 2:**
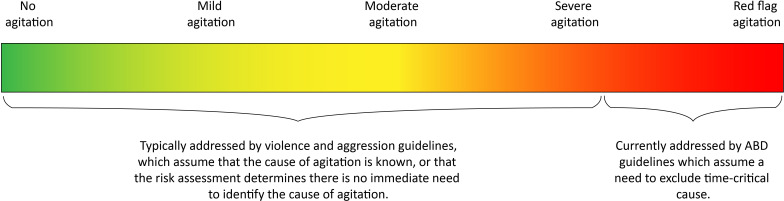
A suggested agitation spectrum applicable across the service user journey. ABD, acute behavioural disturbance.

To build on the consensus in this study, to foster trust with service users, to clearly establish that ABD is of limited utility as a descriptive term and to move towards agitation guidelines which address the entire spectrum of agitation, the consensus features suggested in this study for the recognition of ABD could instead be described as ‘red-flag agitation’ (similar to the highest-risk patients with other conditions such as ‘red-flag sepsis’), identifying patients at highest risk of poor outcomes and most likely to require the most urgent specialised management strategies. A suggested spectrum of agitation is given in figure 2. This spectrum would provide scope for different suggested care strategies across a range of presentations; allow dynamic reassessment and minimise anchoring bias in decision-making; clearly establish that ABD is not a diagnosis; and provide common terminology across prehospital and ED settings regarding prioritisation of care, which would have its foundation in the features identified in this study rather than in historical literature. Additionally, this approach was suggested as a solution by the Royal College of Psychiatrists in their 2022 report on ABD.

### The management of undifferentiated agitation

When people present with severe agitation, healthcare staff may be unable to safely approach the patient and undertake a standard physical health assessment. Clinical investigations and clinical procedures are frequently not possible without sedation. Conversely, sedation is normally delivered in a highly monitored and controlled environment, with prior patient assessment and clinical optimisation. This is the paradox which ABD guidelines have historically tried to address, providing strategies (such as potent intramuscular sedation prior to the application of clinical monitoring) which differ from standard care.

Agitation guidelines will always require adaptation to specific care settings, considering differing expertise, resources, and the level of clinical risk related to unidentified or unmanaged conditions.

### Limitations

There are limitations to the Delphi technique which should be noted. A Delphi study is an iterative process which requires a significant time commitment from participants. While there is a desire to continue repeating rounds until a perfect consensus is achieved, this needs to be balanced against the burden on participants, dropout rates with each round, and the desire from participants and their parent organisations for results within clear timescales. Our analysis of non-consensus statements at the end of round 3 allows a tentative conclusion that stability has been reached, but the value of Krippendorff’s alpha was at the lower limit of the conventional range.

Statements were generated by participants from a broad range of professional backgrounds, with differing perspectives on the aspects of ABD they wished to see represented in the Delphi process. Statements ranged from broad perspectives on the validity of ABD through to specific clinical features denoting high clinical risk.

The scope of the Delphi was deliberately broad and was designed to provide the foundation on which future patient care, guidelines and research could be based, rather than providing immediate resolution for every contentious aspect. Individual organisations who participated may feel that the process has failed to appreciate every nuance of their role in the care of people presenting with ABD. Additionally, it should be noted that it was only possible to vote on statements generated in round 1. This precluded exploration of the implications of statements, reasons for non-consensus and the introduction of additional statements to expand the scope of consensus. One consequence of this is that a small number of contradictory statements emerged from questions 5, 6 and 7. For example, the statement ‘The person has a raised/elevated HR’ was approved for both questions 5 and 6, meaning that this would be included in criteria for an emergency healthcare response (input within 20 min) or an urgent healthcare response (input within 2 hours). In the derived consensus statement, this contradiction was overcome by keeping the 20-minute time frame, which emerged as the only acceptable response to ABD presentations. It should be noted that people with priority symptoms or signs (suggesting a periarrest or cardiac arrest state) would still be prioritised by ambulance services to receive an immediate response.

There were 164 statements which did not achieve consensus, and these largely addressed specific features regarding the identification of ABD. It is possible that a traditional Delphi model including face-to-face discussion would have helped to achieve further consensus. The full list of consensus and non-consensus statements is provided as online supplemental material.

### Next steps

There are key next steps which should be undertaken as a consequence of this study. While they are potentially immediately actionable, this will require funding, formal quality improvement methodology, further research and cross-systems leadership to improve quality of care. Educational materials and clinical guidelines require adaptation to reflect this consensus opinion of UK stakeholder organisations, and remove advice previously given which lacks a meaningful evidence base.

Clinical guidelines should address the full range of presentations of agitation and respective management strategies. Use of the term ABD separately to agitation is not felt to be helpful, but national healthcare and emergency services providers need to agree with any language or terminology changes for first responders, to manage patient safety concerns with regard to consistent language use.

The Delphi criteria will require prospective validation at all points in the service user journey to identify which features perform well in identifying people at risk, identify any other features which have not been identified by this Delphi process and ensure that the established criteria are not subject to bias in their application. The Delphi has provided a consensus against which appropriate or inappropriate management may be measured.

## Conclusion

It is key that ABD should be understood to be a presentation, not a diagnosis. While there are a number of features which may be identified in the highest-risk presentations of agitation, the triad of being hot to touch (tactile hyperthermia), exhibiting constant or near-constant activity, and extreme agitation or aggression should be the focus of recognition.

Specific terminology should be used by first responders to recognise agitated people who require a focus on management of their physical health, and consideration should be given to using new terminology, such as ‘red-flag agitation’ rather than ‘acute behavioural disturbance’ to identify this group of agitated patients at highest risk of poor outcomes, who may require specialised management strategies.

## Data Availability

Data are available upon reasonable request. Data collected for this study, including deidentified participant data (without professional affiliation, to reduce identifiability), will be made available on request to the corresponding author when accompanied by an approved research proposal and an acceptable data access agreement.
