# Supplementary material for: Consensus on acute behavioural disturbance in the UK: a multidisciplinary modified Delphi study to determine what it is and how it should be managed
Source: Emerg Med J. 2023 Sep 22;41(1):4–12. doi: 10.1136/emermed-2023-213335 (PMC10804037; doi:10.1136/emermed-2023-213335)
Supplement: Supplementary data [file emermed-2023-213335supp001.pdf]

Supplementary material

Table 1. Statements achieving consensus in round 2.

Broad question	Statement for voting	Disagree	Agree	Median	Outcome
Question 1	**Statement 1:** All first response professionals should understand that Acute Behavioural Disturbance does not denote a specific diagnosis.	8%	92%	7	agree
Question 1	**Statement 2:** All first response professionals should understand that patients presenting with rapid physical deterioration and agitated behaviour should have a focus on management of their physical health.	8%	88%	7	agree
Question 1	**Statement 3:** There should be a focus on physical health monitoring for evidence of deterioration, use of de-escalation techniques, and early transportation to a healthcare facility if there is evidence of deterioration.	0%	100%	7	agree
Question 1	**Statement 4:** Officers should have a high index of suspicion and therefore a low threshold to divert to a health rather than a custody setting.	4%	92%	7	agree
Question 1	**Statement 5:** The triad of being hot to touch (tactile hyperthermia), exhibiting constant or near constant activity, and extreme agitation or aggression should be the focus of recognition, as the majority of other described signs and symptoms arise as a consequence of these.	4%	88%	6	agree
Question 1	**Statement 11:** The person is hot to touch	13%	79%	6	agree
Question 1	**Statement 15:** The person has a raised heart rate	17%	79%	6	agree
Question 1	**Statement 17:** The person is exhibiting constant physical activity	8%	83%	6	agree
Question 1	**Statement 18:** The person is exhibiting near constant physical activity	4%	96%	6	agree
Question 1	**Statement 19:** The person is exhibiting extreme agitation	4%	96%	6	agree
Question 1	**Statement 20:** The person is exhibiting extreme aggression	13%	79%	6	agree
Question 1	**Statement 34:** The person's presentation of aggression or hostility appears atypical	13%	88%	6	agree
Question 1	**Statement 37:** The person is unable to sit or stand still	17%	75%	5	agree
Question 1	**Statement 57:** Live-feed of officer's body worn video to specifically trained individuals in force control rooms may be helpful.	4%	79%	6	agree
Question 2	**Statement 1:** Custody staff should be trained to recognise a patient who is at high risk of deteriorating early.	4%	96%	7	agree
Question 2	**Statement 2:** Healthcare staff should be aware that restraint may worsen a patient's condition.	4%	96%	7	agree
Question 2	**Statement 3:** It is vital that healthcare staff have plans regarding the management of patients presenting with ABD until emergency care has arrived, including de-escalation.	0%	100%	7	agree
Question 2	**Statement 4:** Emergency equipment should be readily available in environments which may be required to care for people presenting with ABD.	0%	100%	7	agree
Question 2	**Statement 5:** The combination of physical health deterioration and behavioural disorganisation is likely to need emergency medical care.	0%	100%	7	agree
Question 2	**Statement 14:** The person is hot to touch	17%	79%	6	agree
Question 2	**Statement 15:** The person is sweating profusely	17%	75%	5	agree

Question 2	**Statement 18:** The person has a high temperature	8%	92%	6	agree
Question 2	**Statement 25:** The person has a raised heart rate	13%	88%	6	agree
Question 2	**Statement 28:** The person has raised breathing rate and heart rate	13%	75%	6	agree
Question 2	**Statement 29:** The person has autonomic dysfunction	13%	83%	6	agree
Question 2	**Statement 33:** The person is exhibiting constant physical activity	4%	96%	6	agree
Question 2	**Statement 34:** The person is exhibiting near constant physical activity	4%	88%	6	agree
Question 2	**Statement 35:** The person is exhibiting near constant physical activity including aggressive/violent behaviours	0%	96%	6	agree
Question 2	**Statement 37:** The person does not appear to tire	22%	78%	5	agree
Question 2	**Statement 38:** The person is exhibiting extreme agitation	4%	92%	6	agree
Question 2	**Statement 42:** The person is agitated and not interacting or understanding verbal commands	17%	75%	5.5	agree
Question 2	**Statement 43:** The person is exhibiting extreme agitation, which fails to settle with de-escalation techniques, and physiological derangement	8%	88%	6.5	agree
Question 2	**Statement 44:** The person is exhibiting severe agitation with a risk of violence	4%	88%	6	agree
Question 2	**Statement 45:** The person is exhibiting extreme aggression	13%	79%	6	agree
Question 2	**Statement 47:** The person is exhibiting aggression without reason	13%	75%	5	agree
Question 2	**Statement 61:** The person is restrained for 15 minutes without resolution or de-escalation	17%	75%	5	agree
Question 2	**Statement 62:** The person is likely to have ingested stimulant drugs	4%	75%	6	agree
Question 2	**Statement 74:** The person is exhibiting severe delirium	4%	79%	6	agree
Question 2	**Statement 87:** The person appears paranoid	14%	77%	5.5	agree
Question 2	**Statement 88:** The person appears hyper vigilant	13%	79%	5.5	agree
Question 2	**Statement 101:** The person does not respond to de-escalation	8%	75%	5	agree
Question 2	**Statement 117:** Medical / nursing staff have additional skills, training and potentially the opportunity to undertake assessment / screening which could assist in negating the consideration around ABD or better understand it's causation.	8%	75%	6	agree
Question 2	**Statement 118:** Observable features should have slightly more detail for healthcare staff	4%	79%	5.5	agree
Question 3	**Statement 2:** There should be a focus on objective findings: agitation, confusion/disorientation, high anxiety/hyper vigilance/fearfulness/panic, increased physical activity, hot to touch/sweating, tachypnoea, tachycardia.	4%	88%	6	agree

Question 3	**Statement 4:** Healthcare professionals have a responsibility to differentiate the cause of the patient's presentation.	8%	75%	6	agree
Question 3	**Statement 7:** Behaviours that would appear disproportionate to the situation presented (e.g. hyper-arousal)	4%	83%	5	agree
Question 3	**Statement 9:** The person appears severely agitated (unprovoked, or with little provocation)	4%	92%	5.5	agree
Question 3	**Statement 10:** The person appears severely agitated for a prolonged period of time	4%	91%	6	agree
Question 3	**Statement 11:** Police or law enforcement restraint for over 15 minutes	13%	78%	6	agree
Question 3	**Statement 12:** The person has ongoing agitation and is not amenable to verbal or other de-escalation methods	4%	92%	6	agree
Question 3	**Statement 13:** The person has psychomotor agitation	4%	75%	6	agree
Question 3	**Statement 16:** The person has constant or near constant physical activity	4%	88%	6	agree
Question 3	**Statement 17:** The person displays inappropriate behaviour and unresponsiveness to those present	17%	75%	5	agree
Question 3	**Statement 19:** The person is mentally distressed including the potential to harm themselves or another person	8%	79%	5	agree
Question 3	**Statement 23:** The person exhibits an acute deterioration in their condition	0%	96%	6	agree
Question 3	**Statement 24:** The person has an ongoing need for sedation management	4%	92%	6	agree
Question 3	**Statement 27:** The person has abnormal content of consciousness (includes mood, observed behaviour -restlessness, catatonic, agitation etc, and cognition)	21%	79%	6	agree
Question 3	**Statement 32:** The person exhibits a sympathomimetic toxidrome	13%	75%	6	agree
Question 3	**Statement 33:** The person is sweating excessively	8%	88%	5.5	agree
Question 3	**Statement 34:** Temperature (hyperthermia) and associated symptoms such as removal of clothing	4%	83%	6	agree
Question 3	**Statement 35:** The person is hot to touch	4%	88%	6	agree
Question 3	**Statement 36:** The person has a raised respiratory rate	8%	83%	6	agree
Question 3	**Statement 37:** The person has a respiratory rate > 30/min	4%	79%	6	agree
Question 3	**Statement 38:** The person has a raised respiratory rate and increased respiratory effort	13%	75%	6	agree
Question 3	**Statement 39:** The person has a raised pulse rate	13%	83%	5	agree
Question 3	**Statement 40:** The person has a heart rate > 120/min	13%	83%	6	agree
Question 3	**Statement 41:** The person has a heart rate > 150/min	17%	75%	7	agree
Question 3	**Statement 43:** The person has a systolic blood pressure >180-200mmHg	25%	75%	6	agree

Question 3	**Statement 53:** The person has an ongoing need for sedation at higher doses than would typically be expected	8%	88%	6	agree
Question 4	**Statement 1:** A focus on appropriate recognition of ABD (rather than how to discharge patients from ABD guidance) may help prevent this scenario.	4%	87%	6	agree
Question 4	**Statement 2:** If a likely diagnosis or condition is present, then that should be treated, but there may be a role for using ABD guidance in conjunction.	4%	88%	5	agree
Question 4	**Statement 3:** ABD is not a diagnosis, and recognition of a presentation of ABD does not stop other clinical guidelines becoming relevant if a possible, probable or definitive diagnosis is made.	4%	96%	7	agree
Question 4	**Statement 4:** If the patient’s baseline state cannot be determined from personal experience, collateral history, or medical records, the default should be to assume the abnormal behaviour is acute.	4%	92%	6	agree
Question 4	**Statement 5:** It is not helpful to have separate guidance for ‘agitation’ and ‘ABD’. Rather, a history should be taken and assessment of the level of agitation should be made. Treatment should be based on the level of agitation and clinical risk.	8%	75%	5	agree
Question 4	**Statement 6:** Evidence of rapid and significant physical or physiological deterioration along with agitation or confusion should lead to urgent medical assessment and management. Other descriptors are not specific.	4%	96%	6	agree
Question 4	**Statement 7:** Regardless of the terminology used, restraint and sedation should always be a last resort. The first response to any acutely distressed or agitated person should be non-physical approaches, including de-escalation.	0%	100%	7	agree
Question 4	**Statement 17:** The patient is able to communicate their experiences.	9%	78%	5	agree
Question 5	**Statement 1:** When professionals believe the Acute Behavioural Disturbance criteria are met, the person should always be moved to an Emergency Department for assessment, as an emergency	8%	83%	6	agree
Question 5	**Statement 2:** No one sign or symptom should be used in isolation regarding this presentation, the clinical history and preceding events should be used to build a clinical picture	0%	96%	6	agree
Question 5	**Statement 5:** There is a triad of tactile hyperthermia, extreme agitation/aggression, constant/near constant activity	17%	83%	6	agree
Question 5	**Statement 6:** The presentation has been prolonged	17%	79%	5	agree
Question 5	**Statement 8:** Prolonged restraint has been used	13%	83%	6	agree
Question 5	**Statement 9:** The person is restrained for 15 minutes without resolution or de-escalation	13%	87%	6	agree
Question 5	**Statement 10:** If there is continued use of force with a high degree of resistance, with features of ABD	0%	100%	7	agree
Question 5	**Statement 13:** A sudden change in rate or depth of breathing	8%	83%	6.5	agree
Question 5	**Statement 14:** There is behavioural disturbance with clear indication of key drug use, such as cocaine, stimulants, phencyclidine (PCP) or lysergic acid diethylamide (LSD).	4%	83%	6	agree
Question 5	**Statement 16:** There is an inability to safely manage the risk to self or others	8%	92%	7	agree
Question 5	**Statement 17:** The person is a risk to themselves or others	8%	88%	6	agree
Question 5	**Statement 18:** The person has a potential to harm themselves or others	21%	75%	6	agree
Question 5	**Statement 19:** There is persistent agitation	8%	83%	5	agree
Question 5	**Statement 20:** The person is exhibiting extreme agitation	4%	92%	6	agree

Question 5	**Statement 21:** The person is exhibiting constant physical activity	4%	88%	6	agree
Question 5	**Statement 22:** The person is exhibiting near constant physical activity	4%	83%	6	agree
Question 5	**Statement 25:** The person has hyperactivity and agitation that cannot be controlled without medication	9%	91%	6	agree
Question 5	**Statement 26:** The person is exhibiting extreme agitation, which fails to settle with de-escalation techniques, and has caused marked exertion such as active restraint or physiological derangement	4%	92%	6.5	agree
Question 5	**Statement 27:** The person is exhibiting extreme aggression	8%	83%	5.5	agree
Question 5	**Statement 28:** The person is exhibiting aggression and unable to calm with de-escalation	8%	79%	6	agree
Question 5	**Statement 32:** There is abnormality in primary survey (airway, breathing, circulation, disability), abnormal vital signs (using parameters appropriate for age and co-morbidities), abnormal capillary blood glucose or abnormal electrocardiogram	0%	96%	7	agree
Question 5	**Statement 33:** The person has any airway, breathing or circulation problem	4%	92%	7	agree
Question 5	**Statement 35:** The person is sweating profusely	17%	75%	5	agree
Question 5	**Statement 38:** The person has a high temperature	4%	96%	6	agree
Question 5	**Statement 39:** The person has a temperature over 39 degrees Celsius	0%	100%	7	agree
Question 5	**Statement 40:** The person is breathing rapidly	0%	100%	6	agree
Question 5	**Statement 41:** The person is breathing at over 30 breaths per minute	4%	92%	6	agree
Question 5	**Statement 43:** The person has a raised breathing effort/is breathless	8%	79%	6	agree
Question 5	**Statement 44:** The person has a raised heart rate	13%	79%	5	agree
Question 5	**Statement 45:** The person has a heart rate over 120 beats per minute	8%	92%	6	agree
Question 5	**Statement 46:** The person has a heart rate over 150 beats per minute	4%	92%	7	agree
Question 5	**Statement 47:** The person has a heart rate over 160 beats per minute	4%	92%	7	agree
Question 5	**Statement 48:** The person has a systolic blood pressure over 180mmHg	8%	83%	6	agree
Question 5	**Statement 49:** The person has a systolic blood pressure over 200mmHg	8%	88%	7	agree
Question 5	**Statement 50:** The person has clonus	13%	83%	7	agree
Question 5	**Statement 51:** The person has chest pain	8%	92%	7	agree
Question 5	**Statement 52:** The person has low blood pressure	17%	83%	5.5	agree

Question 5	**Statement 53:** The person has a cardiac arrest	0%	96%	7	agree
Question 5	**Statement 54:** The person has a collapse	4%	96%	7	agree
Question 5	**Statement 55:** The person has seizures	0%	96%	7	agree
Question 5	**Statement 56:** The person has low oxygen concentrations	4%	96%	7	agree
Question 5	**Statement 57:** The person has episodes of loss of consciousness	4%	96%	7	agree
Question 5	**Statement 58:** The person has episodes of change of colour	17%	75%	6	agree
Question 5	**Statement 59:** The person has physical injury to self	13%	75%	6	agree
Question 5	**Statement 71:** The person is unresponsive	4%	96%	7	agree
Question 5	**Statement 72:** The person is not responsive to voice	9%	83%	6	agree
Question 5	**Statement 74:** The person has a Glasgow Coma Score below or equal to 8	4%	92%	7	agree
Question 5	**Statement 75:** The person is confused	17%	79%	5.5	agree
Question 5	**Statement 80:** The person has signs of airway obstruction	4%	92%	7	agree
Question 5	**Statement 81:** The person is unable to clear vomitus or secretion	4%	96%	7	agree
Question 5	**Statement 82:** The person has evidence of acute kidney injury, hyperkalaemia, or lactate over 4mmol/L on blood tests	0%	88%	7	agree
Question 6	**Statement 1:** ABD presentations require an emergency response	0%	92%	6	agree
Question 6	**Statement 2:** All patients with a presentation of ABD require urgent healthcare provider input	0%	92%	6	agree
Question 6	**Statement 3:** ABD presentations cannot wait 2 hours for assessment	8%	79%	6	agree
Question 6	**Statement 5:** Fever, prolonged and excessive physical hyperactivity or restraint, episodes of loss of consciousness or change in colour, low oxygen concentrations.	23%	77%	7	agree
Question 6	**Statement 8:** Person presenting with the triad of tactile hyperthermia, extreme agitation, or aggression and near constant physical activity	13%	78%	6	agree
Question 6	**Statement 10:** Person with the presence of physiological hyper arousal along with symptoms of delirium should lead to urgent healthcare input.	13%	87%	6	agree
Question 6	**Statement 23:** Person has an elevated heart rate	22%	78%	5	agree
Question 6	**Statement 24:** Person has a severely high blood pressure	22%	78%	6	agree
Question 6	**Statement 29:** Any deterioration in the person's health	13%	79%	5.5	agree
Question 7	**Statement 1:** ABD presentations require an emergency response.	13%	75%	6	agree

Question 7	**Statement 2:** ABD presentations should always be assessed within 2 hours.	13%	75%	6	agree
Question 7	**Statement 3:** ABD presentations cannot wait 4 hours for assessment.	4%	83%	6.5	agree
Question 7	**Statement 5:** Person who initially presented with high-risk features but has subsequently improved.	4%	83%	5	agree
Question 7	**Statement 6:** Person with mild agitation.	13%	83%	5	agree
Question 7	**Statement 7:** Person able to be settled by family or friends.	9%	87%	6	agree
Question 7	**Statement 14:** Person who has received a normal primary survey (airway, breathing, circulation, disability), normal vital signs (using parameters appropriate for age and co-morbidities), normal capillary blood glucose and a normal electrocardiogram in a patient with previous abnormal behaviour could have a subsequent non-urgent response.	0%	87%	5	agree
Question 8	**Statement 1:** Acute Behavioural Disturbance is always a medical emergency.	17%	78%	6	agree
Question 8	**Statement 9:** Observation in police custody would only be safe following diagnosis of the cause of the ABD and return of temperature, pulse, respiratory rate and other clinical parameters to acceptable levels having been monitored in a setting with full resuscitation facilities.	9%	78%	5	agree
Question 8	**Statement 28:** The person has returned to near baseline and is compliant with interventions.	5%	91%	6	agree
Question 8	**Statement 29:** The person responds to de-escalation.	9%	86%	5	agree
Question 8	**Statement 30:** The person's agitation or violence improves with simple measures	5%	86%	5	agree
Question 8	**Statement 33:** There is decreasing agitation	0%	77%	5	agree
Question 1	**Statement 58:** There is no valid and accepted definition and we therefore cannot describe the features.	87%	13%	2	disagree
Question 2	**Statement 9:** Individual descriptors are insufficient. The person should be exhibiting all consensus features.	75%	13%	2	disagree
Question 2	**Statement 13:** The only features that should be considered are objective and measurable ones such as pulse, temperature, blood pressure and any blood tests possible.	88%	8%	2	disagree
NB: All non-consensus statements entered round 3, and are therefore represented in table 2.					

Table 2. Statement outcomes in round 3.

Broad question	Statement for voting	Disagree	Agree	Median	Outcome
Question 2	**Statement 21:** The person is breathing rapidly	17%	79%	6	agree
Question 2	**Statement 26:** The person has a heart rate over 120 beats per minute	4%	88%	6	agree
Question 2	**Statement 36:** The person has an abnormal level of physical activity	4%	88%	6	agree
Question 2	**Statement 39:** The person is exhibiting agitation	4%	88%	5.5	agree

Question 2	**Statement 40:** The person is unable to calm down	5%	95%	6	agree
Question 2	**Statement 48:** The person is exhibiting extreme violence	0%	87%	6	agree
Question 2	**Statement 49:** The person is exhibiting violent behaviour	4%	75%	5	agree
Question 2	**Statement 56:** The person is constantly resisting restraints	8%	75%	5	agree
Question 2	**Statement 57:** The person is near-constantly resisting restraints	13%	79%	5	agree
Question 2	**Statement 58:** The person has sustained non-compliance with police or ambulance staff	8%	79%	5	agree
Question 2	**Statement 64:** The person is psychotic	8%	75%	5	agree
Question 2	**Statement 65:** The person has symptoms of acute psychosis with fear of impending doom	13%	83%	5	agree
Question 2	**Statement 69:** The features have a sudden onset	4%	83%	5	agree
Question 2	**Statement 72:** The person is exhibiting behaviour that is reportedly not normal for them	4%	92%	6	agree
Question 2	**Statement 73:** The person is exhibiting delirium	4%	88%	6	agree
Question 2	**Statement 75:** The person is unable to sit or stand still	4%	83%	5	agree
Question 2	**Statement 77:** The person has hyper-arousal	0%	92%	6	agree
Question 2	**Statement 83:** The person is disinhibited	4%	83%	5	agree
Question 2	**Statement 84:** The person has a rapid change in behaviour (especially if calm and cooperative previously)	0%	78%	5	agree
Question 2	**Statement 86:** The person appears to be experiencing hallucinations	4%	75%	5	agree
Question 2	**Statement 89:** The person exhibits atypical paranoia	4%	83%	5	agree
Question 2	**Statement 91:** The person appears to be experiencing fear or panic	8%	75%	5	agree
Question 2	**Statement 93:** The person exhibits extreme anxiety	14%	76%	5	agree
Question 2	**Statement 100:** The person does not become calmer with verbal de-escalation	4%	83%	5	agree
Question 2	**Statement 103:** The person has an increased pain threshold/tolerance	4%	78%	5	agree
Question 2	**Statement 113:** The person has a lack of response to first line sedatives	8%	92%	5	agree
Question 2	**Statement 115:** The person has abnormal content of consciousness (includes mood, observed behaviour -restlessness, catatonic, agitation etc, and cognition)	13%	75%	5	agree
Question 2	**Statement 116:** Phrases such as "superhuman strength" should not be used.	9%	87%	6	agree

Question 3	**Statement 1:** The triad of being hot to touch (tactile hyperthermia), exhibiting constant or near constant activity, and extreme agitation or aggression should be the focus of recognition, as the majority of other described signs and symptoms arise as a consequence of these.	0%	92%	6	agree
Question 3	**Statement 3:** As ABD is not a diagnosis, national guidance should be directed towards the safe care of any acutely distressed person in custody.	24%	76%	5	agree
Question 3	**Statement 6:** Being unable to obtain observations (pulse/BP/oximetry/pupil size)	0%	75%	5	agree
Question 3	**Statement 8:** The person appears agitated	0%	75%	5	agree
Question 3	**Statement 15:** The person has an abnormal level of physical activity and/or inability to sit down/settle	4%	88%	6	agree
Question 3	**Statement 18:** The person has ongoing paranoia	4%	79%	5	agree
Question 3	**Statement 25:** The person's presentation is not explained by blood sugar issues	0%	78%	5	agree
Question 3	**Statement 28:** The person has extreme/disproportionate strength	21%	75%	5	agree
Question 3	**Statement 52:** The person does not respond to standard first-line sedative management	4%	87%	5	agree
Question 4	**Statement 12:** The absence of signs or symptoms of physiological disturbance on healthcare staff assessment.	8%	79%	5	agree
Question 4	**Statement 13:** Additional information becomes available (e.g. psychiatric history identifies known schizophrenia) to which symptoms can be attributed.	8%	79%	5	agree
Question 4	**Statement 22:** It is identified that the patient's agitation was mostly provoked (e.g. by unjustifiable restraint or treatment).	0%	92%	5	agree
Question 4	**Statement 23:** The patient is responding to verbal de-escalation.	0%	92%	5	agree
Question 4	**Statement 24:** The person engages in > 60 seconds of consistent verbal communication, or focuses aggression at an individual, demonstrating perception of environment and persons around them.	4%	75%	5	agree
Question 4	**Statement 27:** It is identified that the patient is struggling to breathe	4%	79%	5	agree
Question 5	**Statement 3:** The same criteria should be used as for anyone with acute agitation	8%	84%	5	agree
Question 5	**Statement 11:** A sudden cessation of resistance	0%	96%	6	agree
Question 5	**Statement 12:** The person suddenly becomes quiescent	0%	96%	6	agree
Question 5	**Statement 15:** There is evidence of drug use with a lack of signs of another cause of behavioural disturbance, such as head injury or diabetes.	0%	83%	6	agree
Question 5	**Statement 23:** Prolonged and excessive activity	0%	88%	5.5	agree
Question 5	**Statement 31:** The person is near-constantly resisting restraints	4%	83%	6	agree
Question 5	**Statement 34:** The person is hot to touch	8%	88%	6	agree
Question 5	**Statement 42:** The person is unable to talk in full sentences	4%	83%	6	agree

Question 5	**Statement 63:** The person has any deterioration	8%	92%	6	agree
Question 5	**Statement 64:** The person appears psychotic	8%	88%	6	agree
Question 5	**Statement 66:** The person is exhibiting bizarre behaviour	13%	79%	5	agree
Question 5	**Statement 67:** The person is exhibiting bizarre thoughts	13%	75%	5	agree
Question 5	**Statement 70:** The person has hyper-arousal	0%	83%	5	agree
Question 5	**Statement 77:** The person is making incomprehensible sounds	0%	88%	6	agree
Question 5	**Statement 78:** The person does not respond to de-escalation	4%	83%	5	agree
Question 5	**Statement 83:** The person or circumstances present atypically	4%	75%	5	agree
Question 6	**Statement 6:** Person presenting a risk to self or others	4%	92%	6	agree
Question 6	**Statement 7:** Unable to settle despite basic healthcare measures and reassurance	4%	88%	5	agree
Question 6	**Statement 9:** Person exhibiting behaviours that would appear disproportionate to the situation presented eg hyper arousal	0%	88%	5	agree
Question 6	**Statement 11:** Person with extreme agitation	0%	92%	5	agree
Question 6	**Statement 13:** Person with aggressive behaviour and an inability to remain still and/or calm the person with de-escalation techniques	0%	83%	5	agree
Question 6	**Statement 14:** Person who does not respond to verbal distraction and initial responders feel situation is still high risk/ may escalate	0%	88%	5	agree
Question 6	**Statement 15:** Person with prolonged relentless or extreme physical exertion	0%	88%	5	agree
Question 6	**Statement 16:** Person requiring ongoing restraint and not listening to verbal commands	0%	88%	5	agree
Question 6	**Statement 17:** Person is unable to be deescalated	0%	83%	5	agree
Question 6	**Statement 18:** Person is hot to touch	4%	79%	6	agree
Question 6	**Statement 19:** Person has excessive sweating	8%	83%	5	agree
Question 6	**Statement 21:** Person has constant or near constant physical activity	0%	88%	5	agree
Question 6	**Statement 22:** Person has an increased breathing rate	0%	92%	5	agree
Question 6	**Statement 27:** Person appears disoriented (to place/person/time)	4%	79%	5	agree
Question 6	**Statement 28:** If the person's initial symptoms have not started to resolve	4%	79%	5	agree
Question 7	**Statement 4:** Person without high-risk features who has unresolving symptoms of psychosis, disordered thought content, or confusion.	4%	83%	5	agree

Question 7	**Statement 9:** Person responding coherently to voice.	0%	87%	5	agree
Question 7	**Statement 10:** Person who will sit down.	9%	87%	5	agree
Question 7	**Statement 11:** Person who will eat or drink.	0%	86%	5	agree
Question 8	**Statement 11:** The person's behaviour is proportionate to the situation.	4%	78%	5	agree
Question 8	**Statement 12:** The person remains coherent and able to converse.	0%	87%	5	agree
Question 8	**Statement 13:** The person responds appropriately to verbal command.	4%	83%	5	agree
Question 8	**Statement 14:** The person is able to give rational responses	0%	83%	5	agree
Question 8	**Statement 15:** The person has an absence of any previously described concerning features.	0%	91%	5	agree
Question 8	**Statement 32:** There is only a short period of agitation	4%	83%	5	agree
Question 8	**Statement 34:** The person is not exhibiting paranoia or a high level of agitation	0%	79%	5	agree
Question 8	**Statement 35:** Little or no restraint is used	0%	78%	5	agree
Question 1	**Statement 7:** Individual descriptors are sufficient. The person may only be exhibiting one consensus feature.	83%	4%	3	disagree
Question 1	**Statement 10:** Individual descriptors are insufficient. The person should be exhibiting all consensus features.	100%	0%	2	disagree
Question 1	**Statement 21:** The person is attracted to mirrors or glass	78%	9%	2	disagree
Question 1	**Statement 22:** The person is destroying mirrors or glass	79%	8%	2	disagree
Question 2	**Statement 6:** Individual descriptors are sufficient. The person may only be exhibiting one consensus feature.	88%	4%	3	disagree
Question 2	**Statement 27:** The person has a difficult to obtain wrist pulse	78%	9%	3	disagree
Question 2	**Statement 51:** The person is destroying mirrors or glass	75%	8%	3	disagree
Question 2	**Statement 98:** The person is exhibiting intolerance to light	75%	8%	3	disagree
Question 2	**Statement 105:** The person has a vacant expression	92%	4%	3	disagree
Question 4	**Statement 10:** The decision to move to alternative clinical guidance should only be made in an Emergency Department.	75%	8%	3	disagree
Question 4	**Statement 34:** ABD guidance should not be applied at all, as these presentations are not a separate entity to general agitation.	84%	8%	2	disagree
Question 8	**Statement 4:** There is not an accepted definition of ABD, so this cannot be answered.	76%	16%	2	disagree
Question 1	**Statement 9:** Individual descriptors are insufficient. The person should be exhibiting most consensus features.	17%	46%	4	no consensus

Question 1	**Statement 13:** The person has inappropriately removed clothing	33%	42%	4	no consensus
Question 1	**Statement 14:** The person is breathing rapidly	13%	71%	5	no consensus
Question 1	**Statement 16:** The person has wide dilated pupils	33%	29%	4	no consensus
Question 1	**Statement 23:** The person suddenly becomes quiescent	9%	65%	5	no consensus
Question 1	**Statement 27:** The person expresses a fear of death	46%	25%	4	no consensus
Question 1	**Statement 30:** The person is likely to be exhibiting a mental health presentation	21%	46%	4	no consensus
Question 1	**Statement 31:** The person has symptoms of acute psychosis with fear of impending doom	30%	61%	5	no consensus
Question 1	**Statement 32:** The person is a male age 15-50	21%	21%	4	no consensus
Question 1	**Statement 33:** The person expresses an impending sense of death	33%	25%	4	no consensus
Question 1	**Statement 35:** The person appears irrational	25%	46%	4	no consensus
Question 1	**Statement 38:** The person is unable to engage in conversation	21%	38%	4	no consensus
Question 1	**Statement 39:** The person has bizarre thoughts	33%	33%	4	no consensus
Question 1	**Statement 40:** The person appears to be experiencing hallucinations	29%	38%	4	no consensus
Question 1	**Statement 41:** The person is extremely paranoid	17%	67%	5	no consensus
Question 1	**Statement 42:** The person appears to be experiencing delusions	21%	58%	5	no consensus
Question 1	**Statement 43:** The person is making incomprehensible sounds	21%	50%	4.5	no consensus
Question 1	**Statement 44:** The person's speech is nonsensical	25%	38%	4	no consensus
Question 1	**Statement 45:** The person is exhibiting excessive thirst	33%	21%	4	no consensus
Question 1	**Statement 46:** The person is exhibiting intolerance to light	71%	8%	3	no consensus
Question 1	**Statement 47:** The person is not interacting with responders	48%	22%	4	no consensus
Question 1	**Statement 50:** The person is tolerant of pain	8%	71%	5	no consensus
Question 1	**Statement 53:** The person's behaviour is not explained by a medical condition	17%	42%	4	no consensus
Question 1	**Statement 54:** The person does not appear to be wilfully misbehaving.	25%	38%	4	no consensus
Question 1	**Statement 55:** The patient appears to lack mental capacity (within the meaning of the Mental Capacity Act 2005), as they are unable to (any of):	13%	67%	5	no consensus

	understand information relevant to the decision, retain that information, use or weigh up that information as part of the process of making the decision.				
Question 2	**Statement 8:** Individual descriptors are insufficient. The person should be exhibiting most consensus features.	21%	38%	4	no consensus
Question 2	**Statement 10:** There is insufficient evidence to use 'acute behavioural disturbance' as a unified descriptor.	67%	13%	3	no consensus
Question 2	**Statement 11:** Using features such as unusual strength and lack of tiring increases the risk of racialising acute distress.	71%	17%	3	no consensus
Question 2	**Statement 12:** Using features such as unusual strength and lack of tiring increases the risk of life-threatening restraint situations, particularly for black men.	54%	29%	3	no consensus
Question 2	**Statement 16:** The person has inappropriately removed clothing	8%	58%	5	no consensus
Question 2	**Statement 17:** The person is inappropriately dressed	67%	13%	3	no consensus
Question 2	**Statement 19:** The person has mottled skin	42%	25%	4	no consensus
Question 2	**Statement 20:** The person has excessively red appearing skin	33%	33%	4	no consensus
Question 2	**Statement 22:** The person is breathing at over 30 breaths per minute	8%	71%	6	no consensus
Question 2	**Statement 23:** The person is unable to talk in full sentences	25%	46%	4	no consensus
Question 2	**Statement 24:** The person has a raised breathing effort/is breathless	9%	74%	6	no consensus
Question 2	**Statement 30:** The person has delayed capillary refill time	29%	13%	4	no consensus
Question 2	**Statement 31:** The person has wide dilated pupils	38%	33%	4	no consensus
Question 2	**Statement 32:** The person is restless	13%	58%	5	no consensus
Question 2	**Statement 41:** The person is unable to settle	14%	57%	5	no consensus
Question 2	**Statement 46:** The person is exhibiting aggression	5%	73%	5	no consensus
Question 2	**Statement 50:** The person is attracted to mirrors or glass	67%	8%	3	no consensus
Question 2	**Statement 52:** The person suddenly becomes quiescent	22%	35%	4	no consensus
Question 2	**Statement 53:** The person appears disoriented (to place/person/time)	9%	73%	5	no consensus
Question 2	**Statement 54:** The person is exhibiting exceptional strength	14%	73%	5	no consensus
Question 2	**Statement 55:** The person is exhibiting unexpected strength	17%	63%	5	no consensus
Question 2	**Statement 59:** The person expresses a fear of death	33%	33%	4	no consensus
Question 2	**Statement 60:** The person has a fear of impending doom	33%	46%	4	no consensus

Question 2	**Statement 63:** The person has a history of recent drug use	13%	71%	5.5	no consensus
Question 2	**Statement 66:** The person is a male age 15-50	13%	33%	4	no consensus
Question 2	**Statement 67:** The person expresses an impending sense of death	38%	38%	4	no consensus
Question 2	**Statement 68:** The person or circumstances present atypically	13%	63%	5	no consensus
Question 2	**Statement 70:** The person appears irrational	26%	52%	5	no consensus
Question 2	**Statement 71:** The person is exhibiting bizarre behaviour	9%	61%	5	no consensus
Question 2	**Statement 76:** The person is unable to engage in conversation	13%	39%	4	no consensus
Question 2	**Statement 78:** The person has reduced consciousness	25%	25%	4	no consensus
Question 2	**Statement 79:** The person is not responding to voice	21%	58%	5	no consensus
Question 2	**Statement 80:** The person has a reduction in Glasgow Coma Scale score since presentation	33%	25%	4	no consensus
Question 2	**Statement 81:** The person has incontinence	67%	8%	3	no consensus
Question 2	**Statement 82:** The person is confused	8%	71%	5	no consensus
Question 2	**Statement 85:** The person has bizarre thoughts	4%	61%	5	no consensus
Question 2	**Statement 90:** The person appears catatonic	42%	21%	4	no consensus
Question 2	**Statement 92:** The person appears to be experiencing fear of dogs or being chased	70%	4%	3	no consensus
Question 2	**Statement 94:** The person is making incomprehensible sounds	17%	65%	5	no consensus
Question 2	**Statement 95:** The person's speech is nonsensical	17%	58%	5	no consensus
Question 2	**Statement 96:** The person is exhibiting excessive thirst	25%	46%	4	no consensus
Question 2	**Statement 97:** The person has a dry mouth	29%	17%	4	no consensus
Question 2	**Statement 99:** The person is unresponsive to those present	13%	67%	5	no consensus
Question 2	**Statement 102:** The person is tolerant of pain	8%	71%	5	no consensus
Question 2	**Statement 104:** The person has bizarre or very weak responses to voice, touch or pain.	13%	38%	4	no consensus
Question 2	**Statement 106:** The person has darting or scanning eyes	58%	13%	3	no consensus
Question 2	**Statement 107:** The person has noisy breathing	46%	33%	4	no consensus

Question 2	**Statement 108:** The person has tracheal tug (inward neck movement when breathing)	74%	13%	3	no consensus
Question 2	**Statement 109:** The person has signs of airway obstruction	71%	13%	3	no consensus
Question 2	**Statement 110:** The person has stridor	67%	17%	3	no consensus
Question 2	**Statement 111:** The person has hyper-rigidity	29%	54%	5	no consensus
Question 2	**Statement 112:** The person has signs of a serious condition, such as sepsis, myocardial ischaemia or central neurological system infection.	25%	58%	5	no consensus
Question 2	**Statement 114:** The person has a reduced level of consciousness	39%	22%	4	no consensus
Question 2	**Statement 119:** There should be no significant difference in the initial assessment for police, ambulance, custody healthcare staff or emergency department staff.	67%	17%	3	no consensus
Question 3	**Statement 5:** Healthcare professionals are unlikely to be able to undertake formal clinical examination in presentations of ABD.	13%	71%	5	no consensus
Question 3	**Statement 14:** The person appears hyper vigilant	8%	67%	5	no consensus
Question 3	**Statement 20:** The person shows signs of confusion	8%	71%	5	no consensus
Question 3	**Statement 21:** The person has disordered thought content	13%	71%	5	no consensus
Question 3	**Statement 22:** The person's communicative abilities are compromised	13%	46%	4	no consensus
Question 3	**Statement 26:** The person has a reduced level of consciousness	29%	21%	4	no consensus
Question 3	**Statement 29:** The person exhibits very weak or bizarre response to stimulus	13%	71%	5	no consensus
Question 3	**Statement 30:** The person expresses an impending sense of death	21%	29%	4	no consensus
Question 3	**Statement 31:** The person is inappropriately dressed	30%	26%	4	no consensus
Question 3	**Statement 42:** The person has high blood pressure	8%	58%	5	no consensus
Question 3	**Statement 44:** The person has low blood pressure	38%	13%	4	no consensus
Question 3	**Statement 45:** The person shows signs of dehydration	17%	29%	4	no consensus
Question 3	**Statement 46:** The person's has low blood oxygen levels	38%	21%	4	no consensus
Question 3	**Statement 47:** The person has dilated pupils	17%	42%	4	no consensus
Question 3	**Statement 48:** The person has constricted pupils	46%	17%	4	no consensus
Question 3	**Statement 49:** The person experiences a seizure	17%	71%	5	no consensus
Question 3	**Statement 50:** The person has clonus	17%	71%	6	no consensus

Question 3	**Statement 51:** The person has increased tone	8%	63%	5	no consensus
Question 4	**Statement 8:** The decision to move to alternative clinical guidance should not be made by custody staff.	22%	48%	4	no consensus
Question 4	**Statement 9:** The decision to move to alternative clinical guidance should not be made by ambulance staff.	57%	13%	3	no consensus
Question 4	**Statement 11:** The absence of the previously described signs of ABD on healthcare staff assessment.	9%	43%	4	no consensus
Question 4	**Statement 14:** There is no evidence of acute on chronic stimulant use, nor mental health crisis.	25%	25%	4	no consensus
Question 4	**Statement 15:** The patient’s presentation is improving.	4%	70%	5	no consensus
Question 4	**Statement 16:** The patient’s severe agitation has resolved.	4%	71%	5	no consensus
Question 4	**Statement 18:** The risk of violence has resolved.	8%	67%	5	no consensus
Question 4	**Statement 19:** The patient does not have hyperthermia.	17%	54%	5	no consensus
Question 4	**Statement 20:** The patient recognises they are injured.	21%	25%	4	no consensus
Question 4	**Statement 21:** The patient’s onset of behavioural disturbance is not acute (onset within hours).	13%	38%	4	no consensus
Question 4	**Statement 25:** Female gender makes a presentation of ABD much less likely.	26%	26%	4	no consensus
Question 4	**Statement 26:** The patient’s age affects the likelihood that they are presenting with ABD.	23%	18%	4	no consensus
Question 4	**Statement 28:** It is identified that the patient has chest pain	33%	33%	4	no consensus
Question 4	**Statement 29:** It is identified that the patient has low blood pressure	21%	21%	4	no consensus
Question 4	**Statement 30:** It is identified that the patient has a prolonged capillary refill time	30%	17%	4	no consensus
Question 4	**Statement 31:** A diagnosis of delirium is made	4%	67%	5	no consensus
Question 4	**Statement 32:** It is identified that the patient has incoherent speech	17%	21%	4	no consensus
Question 4	**Statement 33:** It is identified that the patient is exhibiting violent behaviour (as opposed to just agitation)	17%	50%	4.5	no consensus
Question 5	**Statement 4:** There are no features which are robustly predictive of negative outcomes	42%	21%	4	no consensus
Question 5	**Statement 7:** Any restraint has been used	33%	21%	4	no consensus
Question 5	**Statement 24:** The person does not appear to tire	8%	54%	5	no consensus
Question 5	**Statement 29:** The person is exhibiting violent behaviour	0%	71%	5	no consensus
Question 5	**Statement 30:** The person is exhibiting unexpected strength	13%	58%	5	no consensus

Question 5	**Statement 36:** The person has inappropriately removed clothing	29%	33%	4	no consensus
Question 5	**Statement 37:** The person is inappropriately dressed	67%	13%	3	no consensus
Question 5	**Statement 60: The person is attracted to mirrors or glass**	63%	13%	3	no consensus
Question 5	**Statement 61:** The person is destroying mirrors or glass	71%	13%	3	no consensus
Question 5	**Statement 62:** The person expresses a fear of death	29%	29%	4	no consensus
Question 5	**Statement 65:** The person expresses an impending sense of death	17%	54%	5	no consensus
Question 5	**Statement 68:** The person is unable to sit or stand still	8%	71%	5	no consensus
Question 5	**Statement 69:** The person is unable to engage in conversation	13%	29%	4	no consensus
Question 5	**Statement 73:** The person is not listening to verbal commands	0%	33%	4	no consensus
Question 5	**Statement 76:** The person appears paranoid	4%	71%	5	no consensus
Question 5	**Statement 79:** The person is tolerant of pain	21%	33%	4	no consensus
Question 6	**Statement 4:** A patient who remains persistently agitated but there is no exertion or physiological derangement	4%	74%	5	no consensus
Question 6	**Statement 12:** Person who is very aggressive	21%	29%	4	no consensus
Question 6	**Statement 20:** Person inappropriately removes clothing	42%	25%	4	no consensus
Question 6	**Statement 25:** Person is attracted to mirrors or glass	70%	9%	3	no consensus
Question 6	**Statement 26:** Person is destroying mirrors or glass	71%	8%	2.5	no consensus
Question 7	**Statement 8:** Person with a moderately raised temperature.	39%	9%	4	no consensus
Question 7	**Statement 12:** Person who will smoke.	13%	30%	4	no consensus
Question 7	**Statement 13:** Person who has injuries which require assessment or treatment.	30%	35%	4	no consensus
Question 7	**Statement 15:** There are no features which are robustly predictive of negative outcomes.	26%	65%	5	no consensus
Question 7	**Statement 16:** There are no features as we do not have a definition of ABD.	73%	5%	3	no consensus
Question 8	**Statement 2:** If the person begins to settle, this is not a presentation of ABD.	50%	17%	3.5	no consensus
Question 8	**Statement 3:** There are no features known to confer lower risk in ABD.	21%	63%	5	no consensus
Question 8	**Statement 5:** Police custody would not be safe, the person with suspected ABD should always be taken to a healthcare setting.	29%	46%	4	no consensus

Question 8	**Statement 6:** Police custody would only be safe is there is a multidisciplinary decision to ensure safety to the person, or this is part of a monitoring agreement to attempt de-escalation.	30%	30%	4	no consensus
Question 8	**Statement 7:** Police custody would be safe if police are able to regularly observe the person in a custody suite.	46%	29%	4	no consensus
Question 8	**Statement 8:** The person must be assessed in a healthcare setting and confirmation made by a healthcare professional that the person is able to be detained.	22%	70%	5	no consensus
Question 8	**Statement 10:** The person has previous history of the same presentation.	70%	0%	3	no consensus
Question 8	**Statement 16:** The person rests.	9%	39%	4	no consensus
Question 8	**Statement 17:** A National Early Warning Score of zero.	17%	54%	5	no consensus
Question 8	**Statement 18:** The person does not have low oxygen levels.	25%	58%	5	no consensus
Question 8	**Statement 19:** The person does not have periods of loss of consciousness	17%	58%	5	no consensus
Question 8	**Statement 20:** The person does not have a temperature above 38 degrees Celcius	22%	65%	5	no consensus
Question 8	**Statement 21:** The increases in heart rate and/or respiratory rate are mild.	17%	63%	5	no consensus
Question 8	**Statement 22:** The person stops sweating.	17%	25%	4	no consensus
Question 8	**Statement 23:** The person's physical parameters are improving	4%	74%	5	no consensus
Question 8	**Statement 24:** There is an improvement in heart rate.	8%	71%	5	no consensus
Question 8	**Statement 25:** There is an improvement in respiratory rate.	4%	71%	5	no consensus
Question 8	**Statement 26:** There is an improvement in blood pressure.	8%	46%	4	no consensus
Question 8	**Statement 27:** There is an improvement in temperature.	8%	54%	5	no consensus
Question 8	**Statement 31:** The person's severe agitation is transient	8%	71%	5	no consensus
Question 8	**Statement 36:** The person has no restraint related injuries.	17%	48%	4	no consensus
Question 8	**Statement 37:** The person becomes calmer with 2mg or less of lorazepam.	58%	17%	3	no consensus
Question 8	**Statement 38:** The person is willing and able to take oral sedation.	29%	29%	4	no consensus
